# Inferring Amoxicillin-Clavulanate Susceptibility From Ampicillin-Sulbactam Susceptibility Against Common Enterobacterales Species

**DOI:** 10.1093/ofid/ofaf295

**Published:** 2025-05-14

**Authors:** Kimberly C Claeys, Patricia J Simner, Tsigereda Tekle, Anthony D Harris, Sara E Cosgrove, Pranita D Tamma

**Affiliations:** Department of Pharmacy Science and Health Outcomes Research, University of Maryland School of Pharmacy, Baltimore, Maryland, USA; Department of Medicine, Johns Hopkins University School of Medicine, Baltimore, Maryland, USA; Department of Pathology, Johns Hopkins University School of Medicine, Baltimore, Maryland, USA; Department of Pathology, Johns Hopkins University School of Medicine, Baltimore, Maryland, USA; Department of Epidemiology and Public Health, University of Maryland School of Medicine, Baltimore, Maryland, USA; Department of Medicine, Johns Hopkins University School of Medicine, Baltimore, Maryland, USA; Department of Pediatrics, Johns Hopkins University School of Medicine, Baltimore, Maryland, USA

To the  Editor—Transitioning to oral antibiotics to complete treatment courses for invasive Enterobacterales infections is increasingly common [1]. Although fluoroquinolones and trimethoprim-sulfamethoxazole are most frequently employed, both are plagued by notable adverse event profiles [2, 3]. Amoxicillin-clavulanate is an oral β-lactam with high bioavailability and low serum protein binding [4, 5], making it an attractive treatment option that achieves adequate and sustained concentrations in the bloodstream when optimized dosing is employed [6].

The Clinical and Laboratory Standards Institute (CLSI) does not consider ampicillin-sulbactam susceptibility a surrogate for amoxicillin-clavulanate susceptibility [7]. Nonetheless, clinicians commonly use ampicillin-sulbactam susceptibility to infer susceptibility to amoxicillin-clavulanate in the absence of targeted susceptibility testing results for the latter. Although ampicillin can be used to infer susceptibility to amoxicillin given their structural similarity, contemporary investigations into how well the inhibitory activity of sulbactam mimics that of clavulanate are lacking. We sought to investigate if susceptibility of Enterobacterales isolates to ampicillin-sulbactam can be used to predict susceptibility to amoxicillin-clavulanate.

Our study included consecutive *Escherichia coli*, *Klebsiella oxytoca*, *Klebsiella pneumoniae* complex, and *Proteus mirabilis* bloodstream isolates from patients at The Johns Hopkins Hospital from 1 August 2023 to 31 December 2023. Analysis was limited to these 4 species as neither sulbactam nor clavulanate is expected to inhibit organisms with clinically significant AmpC production (eg, *Enterobacter cloacae* complex) [8]. Bacterial species identification was determined by MALDI-TOF MS (Bruker Daltonics Inc). Antimicrobial susceptibility testing of ampicillin-sulbactam and amoxicillin-clavulanate was performed by disk diffusion following CLSI M02 guidelines [9]. Zone diameter measurements to determine susceptibility were in accordance with CLSI recommendations, which were reviewed and unchanged in 2022 [6, 7]. Ampicillin-susceptible isolates were excluded from analysis. Weekly quality control was performed with *E coli* ATCC 35218.

A total of 334 consecutive nonduplicate Enterobacterales bloodstream isolates from unique patients were evaluated. Overall, 212 (63%) isolates were susceptible to ampicillin-sulbactam and 239 (72%) were susceptible to amoxicillin-clavulanate (Table 1).

**Table 1.** Enterobacterales Isolates Susceptible to Ampicillin-Sulbactam and Amoxicillin-Clavulanate by Species

			Isolates Susceptible To		
Species	Total Isolates	Isolates With a *bla*_CTX-M_ Gene	Ampicillin-Sulbactam	Amoxicillin-Clavulanate	Both^a^
*Escherichia coli*	199 (60)	37 (19)	122 (61)	132 (66)	113/122 (93)
*Klebsiella oxytoca*	7 (2)	1 (14)	3 (43)	4 (57)	3/3 (100)
*Klebsiella pneumoniae*	105 (31)	14 (13)	65 (62)	81 (77)	65/65 (100)
*Proteus mirabilis*	23 (7)	2 (9)	22 (96)	22 (96)	22/22 (100)
Total	334 (100)	54 (16)	212 (63)	239 (72)	203/212 (96)

Data are presented as No. (%).

^a^Denominator is based on the ampicillin-sulbactam susceptibility group.

Of the 212 ampicillin-sulbactam susceptible isolates, 203 (96%) were also susceptible to amoxicillin-clavulanate. The 9 isolates susceptible to ampicillin-sulbactam but not to amoxicillin-clavulanate were all *E coli*; 2 of the 9 *E coli* isolates contained a *bla*_CTX-M_ gene. Susceptibility agreement was 100% for *K oxytoca*, *K pneumoniae*, and *P mirabilis.* Susceptibility agreement was 93% for *E coli.*

Ampicillin-sulbactam and amoxicillin-clavulanate became approved by the US Food and Drug Administration in the 1980s and were anticipated to have reliable inhibitory activity against class A β-lactamase enzymes commonly produced by Enterobacterales (eg, SHV-1, TEM-1). Early on, variations in the inactivation chemistry of these inhibitors suggested that there would be differences in their activity against commonly circulating class A β-lactamases [10–12]. For example, the IC50 (ie, a measure of the amount of inhibitor required to decrease enzyme activity to 50%) for clavulanate is 60- and 580-fold lower than sulbactam against TEM-1 and SHV-1, respectively [13]. However, these differences became less apparent over time in large part because the evolution of β-lactamases began to compromise the activity of both agents. Examples of changes reducing the activity of sulbactam and clavulanate include point mutations in *bla*_TEM-1_ and *bla*_SHV-1_ genes resulting in an extended-spectrum β-lactamase phenotype [14], promoter sequence changes leading to increased β-lactamase expression [15], the rapid worldwide dissemination of CTX-M [16], limited affinity against OXA-type enzymes (eg, OXA-1) [17], the presence of AmpC enzymes [8], and the loss of porins reducing β-lactamase inhibitor entry (eg, OmpC, OmpF) [18].

Our results suggest that the relatively unreliable coverage of ampicillin-sulbactam and amoxicillin-clavulanate against Enterobacterales isolates that are nonsusceptible to ampicillin is disappointing at 63% and 72%, respectively, but not surprising, given the remarkable expansion of β-lactamase enzymes since these drugs became clinically available [19]. In our cohort, there was an overall 96% agreement of susceptibility for these 2 agents against common Enterobacterales. Our study suggests that ampicillin-sulbactam–susceptible results can generally be used to infer susceptible results to amoxicillin-clavulanate when amoxicillin-clavulanate results are not readily available, particularly when being considered for urinary tract infections or as “step-down therapy” for invasive infections [6]. This strategy may mitigate the risk of several adverse effects commonly associated with fluoroquinolones—such as *Clostridioides difficile* infection, tendinopathy, neuropsychiatric disturbances, and QT interval prolongation—and trimethoprim-sulfamethoxazole, including hyperkalemia, hypersensitivity reactions, and hematologic cytopenias [2, 20, 21].

## Notes

***Financial support.*** This work was supported through the Patient-Centered Outcomes Research Institute (CER2022C1-26099).

***Potential conflict of interest*.** K. C. C. has served as on advisory boards for bioMérieux. P. J. S. reports receiving grants and personal fees from Accelerate Diagnostics, OpGen, and BD Diagnostics; grants from bioMerieux, Inc, Affinity Biosensors, and Hardy Diagnostics; and personal fees from Roche Diagnostics, Shionogi, Inc, and GeneCapture outside the submitted work. S. E. C. reports receiving personal fees from Debiopharm, outside of the submitted work. All other authors report no potential conflicts.
